# Distributed atomic quantum information processing via optical fibers

**DOI:** 10.1038/s41598-017-01245-x

**Published:** 2017-04-27

**Authors:** Ming-Xing Luo, Hui-Ran Li, Xiaojun Wang

**Affiliations:** 1 Information Security and National Computing Grid Laboratory, Southwest Jiaotong University, Chengdu, 610031 China; 2 Department of Physics, University of Michigan, Ann Arbor, MI 48109 USA; 3 School of Electronic Engineering, Dublin City University, Dublin, 9 Ireland

## Abstract

The qudit system may offer great flexibilities for quantum information processing. We investigate the possibility of realizing elementary quantum gates between two high-dimensional atoms in distant cavities coupled by an optical fiber. We show that highly reliable special swap gate is achievable by different detuning. The numerical simulation shows that the proposed elementary gate is robust against the atomic spontaneous decay, photon leakage of cavities and optical fibers by choosing the experimental parameters appropriately.

## Introduction

Non-classical electromagnetic fields have been described with the quantum mechanical for their special statistical properties, and experimentally realized with quantum optical experiments such as quadrature-squeezed and sub-Poissonian light fields^1–5^. These non-classical light fields may lead various interesting applications such as the enhanced measurement beyond the standard quantum limit set by vacuum fluctuations^4, 6^, or fundamental atomic processes through interaction with non-classical light^7–9^.

A particularly interesting generation of non-classical light fields is related to cavity quantum electrodynamics, in which atoms interact strongly with a single quantized field mode of a cavity^10^. In both the microwave regimes^11–13^ and optical regimes^8, 14, 15^ the single atom cavity mode coupling strength may exceed spontaneous emission and cavity loss rates to produce observable effects of the coupled system. Rydberg atoms^16^ and very high-*Q* superconducting cavities^17^ are constructed in microwave experiments, where spontaneous emission and cavity damping are negligible on the time scale of the atom-field interaction. In optical regimes, the strong-coupling is reached via high-finesse cavities and very small cavity-mode volumes to avoid great spontaneous emission. The optical cavity is convenience because of direct transmission of light through the cavity mirrors, photon counting and homodyne detection^14, 18, 19^.

The coherent evolution makes cavity quantum electrodynamics be favorable candidates for the realizations of photonic Fock states^20, 21^ and Schrödinger cat states^22, 23^. Moreover, by using slowly decaying atomic levels (e.g., Rydberg atoms) or far-off-resonance atom-field interactions, atomic entanglements may be built^24–28^. The realizations of quantum gates between distant qubits in quantum optical settings have been recently investigated^27, 28^. Such proposals are very promising and highly inventive. Serafini *et al*.^29^ investigated the possibility of realizing effective quantum gates between two atoms in distant cavities coupled by an optical fiber. Zheng proposes an efficient scheme for quantum communication between two atoms trapped in distant cavities^30^. Moreover, flying single photons are also used for remote gates^31–35^.

The purpose of this paper is to build the distributed quantum information processing using multilevel atoms. The qudit state (*d*-dimensional state) may offer greater flexibilities for storing quantum information, improving the channel capacity^36, 37^, reducing the implementation complexity of quantum gates^38–41^, enhancing the information security^42–46^ and exploring different quantum features^47–49^. There are various candidate systems for qudit states^50–53^. Unfortunately, few schemes have been proposed for implementing distributed quantum information processing based on qudit systems. Our scheme is based upon the adiabatic transformation of eigenstates of the atom-cavity system^54^. We firstly investigate the possibility of realizing deterministic gates between multi-level atoms in separate optical cavities, through a coherent resonant coupling mediated by an optical fiber. The only control required would be the synchronized switching on and off of the atom-field interactions in the distant cavities, which may be achievable through simple control pulses. Combined with single atomic transformations, the two-atom gate may be used to realize universal qudit quantum logic using recent circuit synthesis^55^. Finally, to show the possibility of these schemes, all the adiabatic conditions are considered. The numerical simulations show that our elementary gates are insensitive to the cavity decay, fiber loss, and atomic spontaneous emission. These gates can be constructed with high fidelity by choosing the parameters appropriately.

## Result

### Remote atomic model

**Figure 1:**
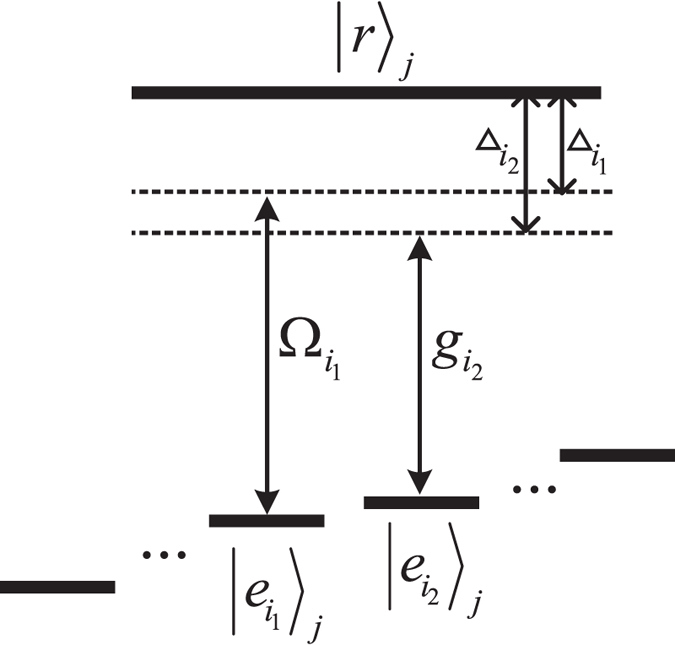
Involved atomic levels and transitions. The transition ${|{e}_{{i}_{1}}\rangle }_{j}\leftrightarrow |r\rangle$ of the *j*-th atom is coupled to the cavity mode with coupling constant *g*_*j*_ and detuning Δ_2_. The transition ${|{e}_{{i}_{2}}\rangle }_{j}\leftrightarrow |r\rangle$ is driven by a classical field with Rabi frequency ${{\rm{\Omega }}}_{j}$ and detuning Δ_1_.

The atomic level configuration is shown in Fig. 1. Each *d* + 1-level atom has an excite state |*r*〉 and *d* ground states $|{e}_{1}\rangle ,\ldots ,|{e}_{d}\rangle$. Two identical multi-level atoms are trapped in distant cavities connected by an optical fiber. The transition $|{e}_{{i}_{2}}\rangle \leftrightarrow |r\rangle$ of each atom is driven by a classical laser field with Rabi frequency ${{\rm{\Omega }}}_{{i}_{1}}$, while the transition $|{e}_{{i}_{1}}\rangle \leftrightarrow |r\rangle$ is driven by the cavity mode with coupling constant ${g}_{{i}_{2}}$. The mode number of the fiber is on the order of $l\overline{\nu }\mathrm{/2}\pi c$, where *l* is the length of the fiber and $\overline{\nu }$ is the decay rate of the cavity field. When $l\overline{\nu }\mathrm{/2}\pi c\le 1$, there is only one fiber mode which essentially interacts with the cavity modes and the cavity-fiber coupling is described by the Hamiltonian as follows^29, 30^

1 $${H}_{0}=\nu b({a}_{1}^{ {\dagger } }+{a}_{2}^{ {\dagger } })+{H}{\rm{.}}{c}{\rm{.}}$$

where *b* is the annihilation operator for the fiber mode, ${a}_{j}^{ {\dagger } }$ is the creation operator for the *j*-th cavity mode, and *ν* is the cavity-fiber coupling strength.

Assume that the classical field and cavity mode are detuned from the respective transition by ${{\rm{\Delta }}}_{{{\rm{i}}}_{{\rm{1}}}}$ and ${{\rm{\Delta }}}_{{i}_{2}}$. In the interaction picture, the Hamiltonian describes the following atom-field interaction

2 $${H}_{int}=\sum _{j=1}^{2}({{\rm{\Omega }}}_{{i}_{1}}{e}^{i{{\rm{\Delta }}}_{{i}_{1}}t}{|r\rangle }_{jj}\langle {e}_{{i}_{1}}|+{g}_{{i}_{2}}{a}_{j}{e}^{i{{\rm{\Delta }}}_{{i}_{2}}t}{|r\rangle }_{jj}\langle {e}_{{i}_{2}}|)+{H}{\rm{.}}{c}{\rm{.}}$$

When ${{\rm{\Delta }}}_{{i}_{1}},{{\rm{\Delta }}}_{{i}_{2}}\gg {{\rm{\Omega }}}_{{i}_{1}},{g}_{{i}_{2}}$, the excite state |*r*〉 can be adiabatically eliminated. It results in the following Hamiltonian

3 $$\begin{array}{rcl}{H}_{int} & = & -\sum _{j=1}^{2}({\eta }_{{i}_{1}}{e}^{i{{\rm{\Delta }}}_{{i}_{1}}t}{|{e}_{{i}_{1}}\rangle }_{jj}\langle {e}_{{i}_{1}}|+{\zeta }_{{i}_{2}}{a}_{j}^{ {\dagger } }{a}_{j}{|{e}_{{i}_{2}}\rangle }_{jj}\langle {e}_{{i}_{2}}|)\\  &  & +\,{\lambda }_{{i}_{1}{i}_{2}}({a}_{j}{S}_{j}^{+}{e}^{i{\delta }_{{i}_{1}{i}_{2}}t}+{a}^{ {\dagger } }{S}_{j}^{-}{e}^{-i{\delta }_{{i}_{1}{i}_{2}}t})+{H}{\rm{.}}{c}{\rm{.}}\end{array}$$

where

4 $${\eta }_{{i}_{1}}=\frac{{{\rm{\Omega }}}_{{i}_{1}}^{2}}{{{\rm{\Delta }}}_{{i}_{1}}},{\zeta }_{{i}_{2}}=\frac{{g}_{{i}_{2}}^{2}}{{{\rm{\Delta }}}_{{i}_{2}}},{\lambda }_{{i}_{1}{i}_{2}}=\frac{{{\rm{\Omega }}}_{{i}_{1}}{g}_{{i}_{2}}}{2}(\frac{1}{{{\rm{\Delta }}}_{{i}_{1}}}+\frac{1}{{{\rm{\Delta }}}_{{i}_{2}}})$$

with ${\delta }_{{i}_{1}{i}_{2}}={{\rm{\Delta }}}_{{i}_{2}}-{{\rm{\Delta }}}_{{i}_{1}}$, ${S}_{j}^{+}={|{e}_{{i}_{1}}\rangle }_{jj}\langle {e}_{{i}_{2}}|$ and ${S}_{j}^{-}={|{e}_{{i}_{2}}\rangle }_{jj}\langle {e}_{{i}_{1}}|$. By introducing new Bosonic modes (see Method), the effective Hamiltonian is reduced to

5 $${H}_{eff}=\sum _{j=1}^{2}{\mu }_{{i}_{1}{i}_{2}}{|{e}_{{i}_{1}}\rangle }_{jj}\langle {e}_{{i}_{1}}|-{\chi }_{{i}_{1}{i}_{2}}({S}_{1}^{+}{S}_{2}^{-}+{S}_{1}^{-}{S}_{2}^{+}),$$

where

6 $${\mu }_{{i}_{1}{i}_{2}}=\frac{{\lambda }_{{i}_{1}{i}_{2}}^{2}}{4}(\frac{2}{{\delta }_{{i}_{1}{i}_{2}}}+\frac{2}{{\delta }_{{i}_{1}{i}_{2}}-\sqrt{2}\nu }+\frac{2}{{\delta }_{{i}_{1}{i}_{2}}+\sqrt{2}\nu })+{\zeta }_{{i}_{1}}.$$

### Distributed qudit computation

It is well-known that the qubit rotations and two-qubit CNOT gate are universal for synthesizing multi-qubit circuit. In this case, one only needs to construct CNOT gate using the system in Fig. 1. In fact, for two three-level atomic systems, each of them has two ground states |*e*_1_〉, |*e*_2_〉, and one excite state |*r*〉. Let atomic transition $|{e}_{2}\rangle \leftrightarrow |r\rangle$ be driven by a classical laser field with Rabi frequency ${\rm{\Omega }}$, while the transition $|{e}_{1}\rangle \leftrightarrow |r\rangle$ be driven by the cavity mode with coupling constant *g*. Assume that the classical field and the cavity mode are detuned from respective transition by Δ_1_ and Δ_2_. In the interaction picture, the Hamiltonian is simplified as

7 $${H}_{eff}=-\mu \sum _{j=1}^{2}{|{e}_{1}\rangle }_{jj}\langle {e}_{1}|+\chi ({S}_{2}^{+}{S}_{3}^{-}+{S}_{2}^{-}{S}_{3}^{+})+H{\rm{.}}c\mathrm{.,}$$

where ${S}_{j}^{+}={|{e}_{1}\rangle }_{jj}\langle {e}_{2}|$ and ${S}_{j}^{-}={|{e}_{2}\rangle }_{jj}\langle {e}_{1}|$. After an evolving time *t*, it leads to a swapping gate

8 $${U}_{2}(\mu ,\chi )=(\begin{array}{cccc}{e}^{-i2\mu t} & 0 & 0 & 0\\ 0 & {e}^{-i\mu t}\,\cos (\chi t) & i{e}^{-i\mu t}\,\sin (\chi t) & 0\\ 0 & i{e}^{-i\mu t}\,\sin (\chi t) & {e}^{-i\mu t}\,\cos (\chi t) & 0\\ 0 & 0 & 0 & 1\end{array}).$$

Moreover, when $\mu t=\mathrm{(2}k+\mathrm{1)}\pi$ and $\chi t=\mathrm{(2}s+\mathrm{1/2)}\pi$ for some integers *k* and *s*, it reduces to the special SWAP gate

9 $$iSWAP=(\begin{array}{cccc}1 & 0 & 0 & 0\\ 0 & 0 & -i & 0\\ 0 & -i & 0 & 0\\ 0 & 0 & 0 & 1\end{array}).$$

**Figure 2:**
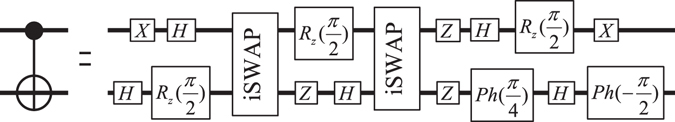
The circuit decomposition of the CNOT gate using the iSWAP gate and single qubit gates. *H* denotes the Hadamard gate. *Z* denotes the Pauli phase flip gate. *R*_*z*_(*θ*) denotes the rotation along the *z*-axis on the Bloch sphere with the angle *θ* while *Ph*(*θ*) denotes the global phase gate with angle *θ*.

This gate may be used to generate CNOT gate, as shown in Fig. 2.

#### Qudit case

Now, we consider the qudit-based quantum computation. From previous result^55^, the set of qudit gates {*C*_2_[*X*_*d*_], *X*_*d*_} is universal for synthesizing multi-qudit circuits. Here, *X*_*d*_ denotes the single qudit operation of *R*_*ij*_(*θ*) or *Z*_*d*_ with the following forms

10 $$\begin{array}{rcl}{R}_{ij}(\theta ) & = & (\begin{array}{ccccc}{I}_{i-1} &  &  &  & \\  & \cos (\theta ) & \cdots  & \sin (\theta ) & \\  & \vdots  & \ddots  & \vdots  & \\  & -\sin (\theta ) & \cdots  & \cos (\theta ) & \\  &  &  &  & {I}_{d-j}\end{array}),\\ {Z}_{d}(\theta ) & = & (\begin{array}{ccccc}{e}^{i{\varphi }_{1}} &  &  &  & \\  & {e}^{i{\varphi }_{2}} &  &  & \\  &  & \ddots  &  & \\  &  &  & {e}^{i{\varphi }_{d-1}} & \\  &  &  &  & {e}^{i{\varphi }_{d}}\end{array}),\end{array}$$

and *C*_2_[*X*_*d*_] denotes the controlled qudit operation defined by

11 $${C}_{2}[{X}_{d}]=(\begin{array}{cc}{I}_{{d}^{2}-d} & \\  & {X}_{d}\end{array}).$$

**Figure 3:**
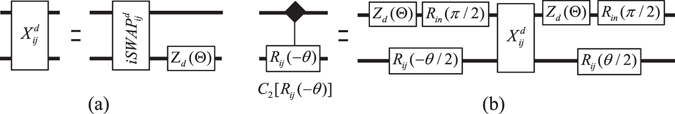
(**a**) The circuit decomposition of two-qudit gate ${X}_{ij}^{d}$ defined in Eq. (15). (**b**) The circuit decomposition of two-qudit elementary gate *C*_2_[*R*_*ij*_(*θ*)]. ${R}_{ij}^{\ast }={R}_{ij}(\pi \mathrm{/2)}{Z}_{d}(-{\rm{\Theta }})$, where ${Z}_{d}(-{\rm{\Theta }})$ is defined in Eq. (16) with ${\rm{\Theta }}=({0}_{d-1},\pi )$.

Since the qudit gate *X*_*d*_ may be realized assisted by the classical fields^54^. In the follow, our consideration is to realize *C*_2_[*X*_*d*_] with the proposed atomic systems in Fig. 1. Firstly, we consider *C*_2_[*R*_*ij*_] with two *d* + 1-level atoms. Two cavity modes are coupled to the transition $|{e}_{i}\rangle \leftrightarrow |r\rangle$ of two atoms with the same detuning Δ_1_. The transition $|{e}_{j}\rangle \leftrightarrow |r\rangle$ of two atoms is driven by classical fields with the same coupling coefficient ${\rm{\Omega }}$ and detuning Δ_2_. In this case, the effective Hamiltonian is simplified as

12 $${H}_{eff}=-\mu ({|{e}_{i}\rangle }_{11}\langle {e}_{i}|+{|{e}_{i}\rangle }_{22}\langle {e}_{i}|)+\chi ({S}_{2}^{+}{S}_{3}^{-}+{S}_{2}^{-}{S}_{3}^{+})+H{\rm{.}}c\mathrm{.,}$$

where ${S}_{k}^{+}={\langle {e}_{j}|}_{kk}\langle {e}_{i}|$ and ${S}_{k}^{-}={|{e}_{i}\rangle }_{kk}\langle {e}_{j}|$. After a proper evolving time *t* (*μt* = (2*k* + 1)*π* and $\chi t=\mathrm{(2}s+\mathrm{1/2)}\pi$ for some integers *k* and *s*), it leads to a special swapping gate as follows:

13 $$iSWA{P}_{ij}^{d}=-i|{e}_{i}{e}_{j}\rangle \langle {e}_{j}{e}_{i}|-i|{e}_{j}{e}_{i}\rangle \langle {e}_{i}{e}_{j}|+{i}{\rm{.}}{d}{\rm{.}}{t}.,$$

where *i*.*d*.*t* denotes the identity operation for all the other terms except to |*e*_*i*_ *e*_*j*_〉 and |*e*_*j*_ *e*_*i*_〉 of two atoms. From the circuit in Fig. 3(a), it easily follows that

14 $${X}_{ij}^{d}=({I}_{d}\otimes {Z}_{d}({\rm{\Theta }}))\times iSWA{P}_{ij}^{d},$$

where ${X}_{ij}^{d}$ is defined by

15 $${X}_{ij}^{d}=|{e}_{i}{e}_{i}\rangle \langle {e}_{i}{e}_{j}|+|{e}_{i}{e}_{j}\rangle \langle {e}_{i}{e}_{i}|+i{\rm{.}}d{\rm{.}}t,$$

and *i*.*d*.*t* denotes the identity operation for all the other terms except to |*e*_*i*_ *e*_*i*_〉 and |*e*_*i*_ *e*_*j*_〉 of two atoms, and ${\rm{\Theta }}=({0}_{i-1},\pi \mathrm{/2},{0}_{j-i-1},\pi \mathrm{/2},{0}_{d-j-1})$ with 0_*k*_ being a zero vector of *k*-dimension.

The two-qudit gate ${X}_{ij}^{d}$ may be used to realize controlled qudit gate *C*_2_[*X*_*d*_]. From Fig. 3(b), note that

16 $$\begin{array}{rcl}{C}_{2}[{R}_{ij}(-\theta )] & = & ({I}_{d}\otimes {R}_{ij}(\frac{\theta }{2}))\times ({R}_{in}(\frac{\pi }{2}){Z}_{d}({\rm{\Theta }})\otimes {I}_{d})\times {X}_{ij}^{d}\\  &  & \times \,({R}_{in}(\frac{\pi }{2}){Z}_{d}({\rm{\Theta }})\otimes {I}_{d})\times ({I}_{d}\otimes {R}_{ij}(\frac{-\theta }{2})),\end{array}$$

where ${\rm{\Theta }}=({0}_{d-1},\pi )$. Now, for an elementary two-qudit gate ${C}_{2}[{Z}_{d}({\rm{\Theta }})]$, from each ${\rm{\Theta }}=({\theta }_{1},{\theta }_{2},\ldots ,{\theta }_{d})$, ${C}_{2}[{Z}_{d}({\rm{\Theta }})]$ may be decomposed into special two-qudit gates as follows

17 $${C}_{2}[{Z}_{d}({\rm{\Theta }})]=\prod _{(i,j)\in {\mathscr{S}}}{C}_{2}[{Z}_{d}({{\rm{\Theta }}}_{ij})],$$

where ${{\rm{\Theta }}}_{ij}=({0}_{i-1},\,{\theta }_{i}{\mathrm{,0}}_{j-i-1},\,{\theta }_{j}{\mathrm{,0}}_{d-j-1})$, and ${\mathscr{S}}$ denotes the integer-pair partition of the index set $\{1,2,\ldots ,d\}$. Now for simplicity, consider the subspace defined by $\{|{e}_{i}{e}_{i}\rangle ,|{e}_{i}{e}_{j}\rangle ,|{e}_{j}{e}_{i}\rangle ,|{e}_{j}{e}_{j}\rangle \}$ while the other subspace is unchanged for the following evaluations. From the Hamiltonian *H*_*eff*_ in Eq. (12), after a proper evolution time *t* ($\chi t=2k\pi$), it follows a two-qudit rotation

18 $$C{Z}_{d}\mathrm{(2}\varphi ,\varphi ,\varphi ,0):={e}^{-i2\mu t}|{e}_{i}{e}_{i}\rangle \langle {e}_{i}{e}_{i}|+{e}^{-i\mu t}|{e}_{i}{e}_{j}\rangle \langle {e}_{i}{e}_{j}|+{e}^{-i\mu t}|{e}_{j}{e}_{i}\rangle \langle {e}_{j}{e}_{i}|+i{\rm{.}}d{\rm{.}}t\mathrm{.}$$

with $\varphi =-\mu t$. From Eqs (10) and (18), it follows that

19 $$\begin{array}{lll}C{Z}_{d}\mathrm{(2}\varphi ,\varphi ,\pi ,\,\varphi ) & := & ({R}_{jn}(\frac{\pi }{2})\otimes {I}_{d})\times C{Z}_{d}\mathrm{(2}\varphi ,\varphi ,0,\varphi )\\  &  & \times \,{C}_{2}[{R}_{ij}(-\frac{\pi }{2})]\times ({R}_{jn}(\frac{\pi }{2})\otimes {I}_{d})\\  & = & {e}^{-i2\mu t}|{e}_{i}{e}_{i}\rangle \langle {e}_{i}{e}_{i}|+{e}^{-i\mu t}|{e}_{i}{e}_{j}\rangle \langle {e}_{i}{e}_{j}|\\  &  & -|{e}_{j}{e}_{i}\rangle \langle {e}_{j}{e}_{i}|+{e}^{-i\mu t}|{e}_{j}{e}_{j}\rangle \langle {e}_{j}{e}_{j}|+i{\rm{.}}d{\rm{.}}t\mathrm{.}\end{array}$$

Similarly, one can get

20 $$\begin{array}{lll}C{Z}_{d}(\varphi +\pi ,\,2\varphi ,\,\varphi ,\,\mathrm{0)} & := & ({R}_{in}(\frac{\pi }{2})\otimes {I}_{d})\times C{Z}_{d}\mathrm{(2}\varphi ,\varphi ,\pi ,\varphi )\\  &  & \times \,{C}_{2}[{R}_{ij}(-\frac{\pi }{2})]\times ({R}_{in}(\frac{\pi }{2})\otimes {I}_{d})\\  & = & {e}^{i(\pi -\mu t)}|{e}_{i}{e}_{i}\rangle \langle {e}_{i}{e}_{i}|+{e}^{-i2\mu t}|{e}_{i}{e}_{j}\rangle \langle {e}_{i}{e}_{j}|\\  &  & +\,{e}^{-i\mu t}|{e}_{j}{e}_{i}\rangle \langle {e}_{j}{e}_{i}|+i{\rm{.}}d{\rm{.}}t\mathrm{.}\end{array}$$

Two phase gates yield to

21 $$\begin{array}{lll}C{Z}_{d}\mathrm{(3}\varphi +\pi ,3\varphi ,\varphi +\pi ,\varphi ) & := & C{Z}_{d}(\varphi +\pi ,2\varphi ,\varphi ,0)\cdot C{Z}_{d}\mathrm{(2}\varphi ,\varphi ,\pi ,\varphi )\\  & = & {e}^{i(-3\mu t+\pi )}|{e}_{i}{e}_{i}\rangle \langle {e}_{i}{e}_{i}|+{e}^{-i3\mu t}|{e}_{i}{e}_{j}\rangle \langle {e}_{i}{e}_{j}|\\  &  & +\,{e}^{i(\pi -\mu t)}|{e}_{j}{e}_{j}\rangle \langle {e}_{j}{e}_{j}|+{e}^{-i\mu t}|{e}_{j}{e}_{i}\rangle \langle {e}_{j}{e}_{i}|+i{\rm{.}}d{\rm{.}}t\mathrm{.}\end{array}$$

From Eqs (10) and (21), it follows that

22 $$\begin{array}{lll}C{Z}_{d}\mathrm{(3}\varphi ,3\varphi ,\varphi ,\varphi ) & := & ({R}_{jn}(\frac{\pi }{2})\otimes {I}_{d})\times ({R}_{in}(\frac{\pi }{2})\otimes {I}_{d})\\  &  & \times \,C{Z}_{d}\mathrm{(3}\varphi +\pi ,3\varphi ,\varphi +\pi ,\varphi )\times {C}_{2}[{R}_{ij}(\frac{\pi }{2})]\\  &  & \times \,({R}_{in}(\frac{\pi }{2})\otimes {I}_{d})\times {C}_{2}[{R}_{ij}(\frac{\pi }{2})]\times ({R}_{in}(\frac{\pi }{2})\otimes {I}_{d})\\  & = & {e}^{-i3\mu t}|{e}_{i}{e}_{i}\rangle \langle {e}_{i}{e}_{i}|+{e}^{-i3\mu t}|{e}_{i}{e}_{j}\rangle \langle {e}_{i}{e}_{j}|\\  &  & +{e}^{-i\mu t)}|{e}_{j}{e}_{j}\rangle \langle {e}_{j}{e}_{j}|+{e}^{-i\mu t}|{e}_{j}{e}_{i}\rangle \langle {e}_{j}{e}_{i}|+i{\rm{.}}d{\rm{.}}t\mathrm{.}\end{array}$$

From Eqs (10) and (22), it follows that

23 $$\begin{array}{lll}C{Z}_{d}(\varphi ,\varphi ,3\varphi ,3\varphi ) & := & ({Z}_{d}({\rm{\Theta }}){R}_{ij}(\frac{\pi }{4}){R}_{ij}(\frac{\pi }{4})\otimes {I}_{d})\\  &  & \times \,C{Z}_{d}\mathrm{(3}\varphi ,3\varphi ,\varphi ,\varphi )\times ({Z}_{d}({\rm{\Theta }}){R}_{ij}(\frac{\pi }{4}){R}_{ij}(\frac{\pi }{4})\otimes {I}_{d})\\  & = & {e}^{-i\mu t}|{e}_{i}{e}_{i}\rangle \langle {e}_{i}{e}_{i}|+{e}^{-i\mu t}|{e}_{i}{e}_{j}\rangle \langle {e}_{i}{e}_{j}|\\  &  & +\,{e}^{-i3\mu t)}|{e}_{j}{e}_{j}\rangle \langle {e}_{j}{e}_{j}|+{e}^{-i3\mu t}|{e}_{j}{e}_{i}\rangle \langle {e}_{j}{e}_{i}|+i{\rm{.}}d{\rm{.}}t\mathrm{.}\end{array}$$

where ${\rm{\Theta }}=({0}_{j-1},\pi ,{0}_{d-j})$. From Eqs (27) and (28), we obtain

24 $$\begin{array}{lll}C{Z}_{d}\mathrm{(4}\varphi ,4\varphi ,4\varphi ,4\varphi ) & := & C{Z}_{d}(\varphi ,\varphi ,3\varphi ,3\varphi )C{Z}_{d}\mathrm{(3}\varphi ,3\varphi ,\varphi ,\varphi )\\  & = & {e}^{-i4\mu t}|{e}_{i}{e}_{i}\rangle \langle {e}_{i}{e}_{i}|+{e}^{-i4\mu t}|{e}_{i}{e}_{j}\rangle \langle {e}_{i}{e}_{j}|\\  &  & +\,{e}^{-i4\mu t)}|{e}_{j}{e}_{j}\rangle \langle {e}_{j}{e}_{j}|+{e}^{-i4\mu t}|{e}_{j}{e}_{i}\rangle \langle {e}_{j}{e}_{i}|+i.d.t.,\end{array}$$

25 $$\begin{array}{lll}C{Z}_{d}\mathrm{(2}\varphi ,2\varphi ,-2\varphi ,-2\varphi ) & := & C{Z}_{d}\mathrm{(3}\varphi ,3\varphi ,\varphi ,\varphi )C{Z}_{d}(-\varphi ,-\varphi ,-3\varphi ,-3\varphi )\\  & = & {e}^{-i2\mu t}|{e}_{i}{e}_{i}\rangle \langle {e}_{i}{e}_{i}|+{e}^{-i2\mu t}|{e}_{i}{e}_{j}\rangle \langle {e}_{i}{e}_{j}|\\  &  & +\,{e}^{i2\mu t)}|{e}_{j}{e}_{j}\rangle \langle {e}_{j}{e}_{j}|+{e}^{i2\mu t}|{e}_{j}{e}_{i}\rangle \langle {e}_{j}{e}_{i}|+i\mathrm{.}d\mathrm{.}t\mathrm{.},\end{array}$$

where $C{Z}_{d}(-\varphi ,\,-\varphi ,\,-3\varphi ,\,-3\varphi )$ may be obtained by letting $-\varphi =2\pi -\varphi =-\mu t$ for some *t*. Therefore, Eqs (23) and (25) lead to

26 $$\begin{array}{lll}C{Z}_{d}\mathrm{(6}\varphi ,6\varphi ,0,\mathrm{0)} & := & C{Z}_{d}\mathrm{(2}\varphi ,2\varphi ,-2\varphi ,-2\varphi )C{Z}_{d}\mathrm{(4}\varphi ,4\varphi ,4\varphi ,4\varphi )\\  & = & {e}^{-i6\mu t}|{e}_{i}{e}_{i}\rangle \langle {e}_{i}{e}_{i}|+{e}^{-i6\mu t}|{e}_{i}{e}_{j}\rangle \langle {e}_{i}{e}_{j}|+i{\rm{.}}d{\rm{.}}t\mathrm{.}\end{array}$$

Finally, the gate ${C}_{2}[{Z}_{d}({{\rm{\Theta }}}_{ij})]$ may be realized from the decomposition of $({R}_{in}(\pi \mathrm{/2)}\otimes {I}_{d})\times C{Z}_{ij}\times ({R}_{in}(\pi \mathrm{/2})\otimes {I}_{d})$ for different *ϕ*.

### Effects of spontaneous decay and photon leakage

In this section, we study the influence of atomic spontaneous decay and photon leakage of the cavities and fibers. For convenience, we rewrite the interaction Hamiltonian under the dipole and rotating wave approximation. The master equation for the density matrices of the system is expressed as

27 $$\begin{array}{rcl}\dot{\rho } & = & -i[H,\rho ]-\sum _{j}\{\frac{{\kappa }_{{f}_{j}}}{2}({b}_{j}^{ {\dagger } }{b}_{j}\rho -2{b}_{j}\rho {b}_{j}^{ {\dagger } }+\rho {b}_{j}^{ {\dagger } }{b}_{j})\\  &  & +\,\frac{{\kappa }_{{c}_{j}}}{2}({a}_{j}^{ {\dagger } }{a}_{j}\rho -2{a}_{j}\rho {a}_{j}^{ {\dagger } }+\rho {a}_{j}^{ {\dagger } }{a}_{j})\\  &  & +\,\sum _{i=1}^{d}\frac{{\gamma }_{j}^{ri}}{2}({\sigma }_{j}^{rr}\rho -2{\sigma }_{j}^{ir}\rho {\sigma }_{j}^{rx}+\rho {\sigma }_{j}^{rr})\end{array}$$

where ${\kappa }_{{f}_{j}}$ and ${\kappa }_{{c}_{j}}$ denote the decay rates of the *j*-th cavity field and the *j*-th fiber mode, respectively, ${\gamma }_{j}^{rx}$ denotes the spontaneous decay rate of the *j*-th atom from level |*r*〉 to |*e*_*i*_〉, and ${\sigma }_{k}^{js}={|j\rangle }_{kk}\langle s|(j,s=1,\ldots ,d)$ are the usual Pauli matrices. For the convenience, assume that ${\kappa }_{{f}_{j}}={\kappa }_{{c}_{s}}=\kappa$ and ${\gamma }_{j}^{rx}={\gamma }_{a}/d$ due to the equal probability transition of $|r\rangle \leftrightarrow |{e}_{i}\rangle$. In the follow, we will discuss the parameter conditions and experimental feasibility of the present scheme. With the choice of a scaling *g*, all the parameters can be reduced to the dimensionless units related to *g*.

**Figure 4:**
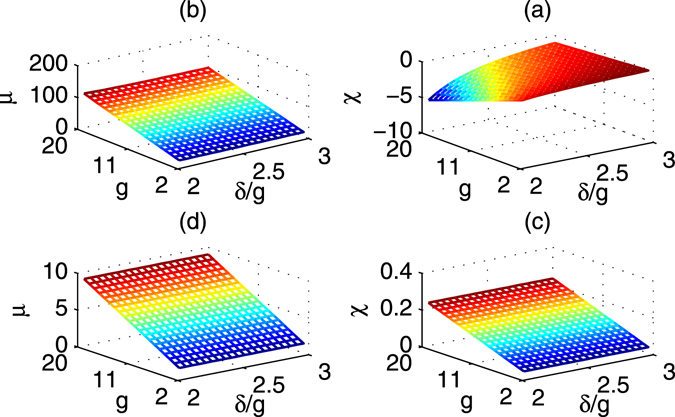
Two phase parameters *μ* and *χ* vias relative detuning δ/*g* and coupling strength *g*. (**a**) *χ* vias *δ*/*g* and *g*. (**b**) *μ* vias *δ*/*g* and *g*. Here, Δ_1_ = 4*g*, Δ_2_ = 4*g* + *δ*, *ν* = *g*, ${\rm{\Omega }}=g$. *μ* > 0 and *χ* < 0 are useful for generating negative phases. (**c**) *χ* vias *δ*/*g* and *g*. (**d**) *μ* vias *δ*/*g* and *g*. Here, Δ_1_ = 9*g*, Δ_2_ = 9*g* + *δ*, *ν* = 4*g*, ${\rm{\Omega }}=3g$. Here, *χ* and *μ* are positive.

**Figure 5:**
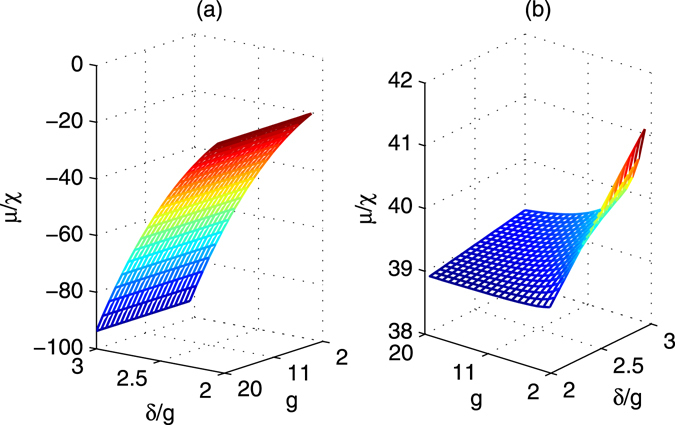
*μ*/*χ* vias *δ*/*g* and *g*. (**a**) Δ_1_ = 4*g*, Δ_2_ = 4*g* + *δ*, *ν* = *g*, $${\rm{\Omega }}=g$$; (**b**) Δ_1_ = 9*g*, Δ_2_ = 9*g* + *δ*, *ν* = 4*g*, $${\rm{\Omega }}=3g$$.

To realize various rotations in Eqs (9) and (15), the rotation parameters *χ* and *μ* could achieve various values. In detail, consider the parameters of Δ_1_ = 4*g*, Δ_2_ = 4*g* + *δ*, *ν* = *g* and ${\rm{\Omega }}=3g$. The rotation parameters *χ* and *μ* are shown in Fig. 4(a,b) respectively. It follows that *μ* may be changed largely while *χ* is negative. The ratio of *μ* and *χ* is changed from −110 to −20 in Fig. 5(a). Moreover, if another set of parameters Δ_1_ = 9*g*, Δ_2_ = 9*g* + *δ*, *ν* = 4*g* and ${\rm{\Omega }}=3g$ are considered, the rotation parameters *χ* and *μ* are shown in Fig. 4(c,d) respectively. In this case, both of them are positive where their ratio is shown in Fig. 5(b).

**Figure 6:**
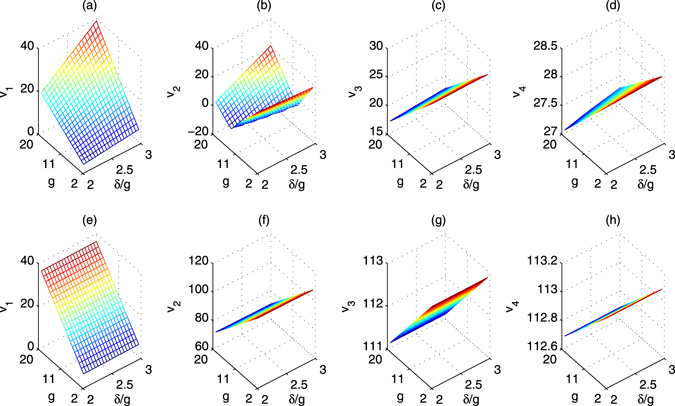
The adiabatic conditions vias *δ*/*g* and *g*. (**a**–**d**) Denote the first case in Fig. 4. (**e**–**h**) Denote the second case in Fig. 4. Here, *v*_1_ = *δ* − *λ*, ${v}_{2}=|\delta -\sqrt{2}\nu |-\lambda /2$, ${v}_{3}=\sqrt{2}\nu -\lambda /2$, and ${v}_{4}=\sqrt{2}\nu -\eta /4$.

For the first set of parameters shown in Fig. 4(a), all the adiabatic conditions ${v}_{i}\gg 0$ of *v*_1_ = *δ* − *λ*, ${v}_{2}=|\delta -\sqrt{2}\nu |-\lambda \mathrm{/2}$, ${v}_{3}=\sqrt{2}\nu -\lambda \mathrm{/2}$, and ${v}_{4}=\sqrt{2}\nu -\eta \mathrm{/4}$ are approximatively satisfied when *g* and *δ*/*g* are increased, as shown in Fig. 6(a–d). Here, *v*_2_ < 0 should be avoided by choosing proper *g* and *δ*. If the second set of parameters shown in Fig. 4(c) are considered, the corresponding adiabatic conditions ${v}_{i}\gg 0$ of are greatly improved and shown in Fig. 6(f–h). Specially, in this case, all the *v*_*i*_ > 0 for all *g* > 2 and *δ*/*g* > 2. It means that the adiabatic conditions may be satisfied under the weak coupling *g* < 5.

**Figure 7:**
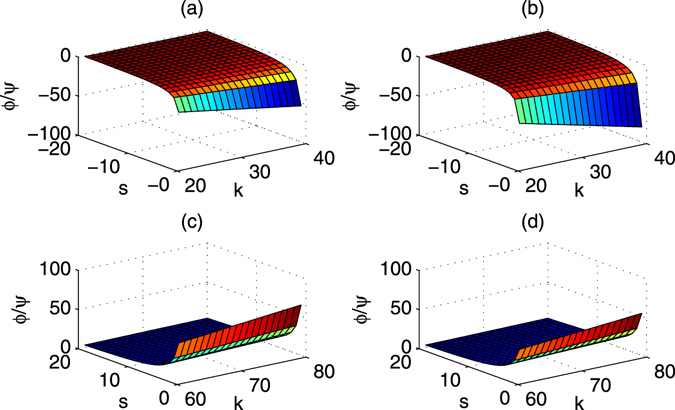
The phase ratio $\varphi /\psi =(\mu t)/(\chi t)$ for the iSWAP gate and the inverse of iSWAP. (**a**,**b**) Denote the evolution times using the first set of parameters shown in Fig. 4(a). (**c**,**d**) Denote the evolution times using the second set of parameters shown in Fig. 4(c).

In order to complete the quantum applications, proper quantum gates should be realized using special phases *ϕ* = *μt* and $\psi =\chi t$ with proper evolution times. The phases ratio $\varphi /\psi$ of all the gates including the iSWAP gate and inverse iSWAP gate are shown in Fig. 7(a,b). Combined with Fig. 5(a), these gates may be efficiently realized. Moreover, if another set of parameters Δ_1_ = 9*g*, ${{\rm{\Delta }}}_{2}=9g+\delta$, $\nu =4g$, and ${\rm{\Omega }}=3g$ are considered, the rotation parameters *χ* and *μ* are shown in Fig. 4(c,d) respectively. In this case, both of them are positive, and their ratio is shown in Fig. 5(b). The corresponding adiabatic conditions are improved and shown in Fig. 6(e,f). The phases ratio $\varphi /\psi$ of different gates are shown in Fig. 7(c,d), which mean that the iSWAP gate and inverse iSWAP gate may be realized.

To consider atomic spontaneous emission and the decay of the Bosonic modes, let ${\rm{\Gamma }}=\kappa =\gamma =0.01g$, where Γ, *κ*, and *γ* are the decay rates for the atomic excited state, the cavity modes, and the fiber mode, respectively. The probability that the atoms undergo a transition to the excited state due to the off-resonant interaction with the classical fields is ${P}_{1}={\rm{\Gamma }}/{{\rm{\Delta }}}_{1}^{2} < 0.01$ for both cases. Meanwhile, the probability that the three modes *c*_*i*_ are excited due to non-resonant coupling with the classical modes is

28 $${P}_{2}\approx \frac{{\lambda }^{2}}{4}[\frac{2}{{\delta }^{2}}+\frac{1}{{(\delta -\sqrt{2}\nu )}^{2}}+\frac{1}{{(\delta +\sqrt{2}\nu )}^{2}}].$$

**Figure 8:**
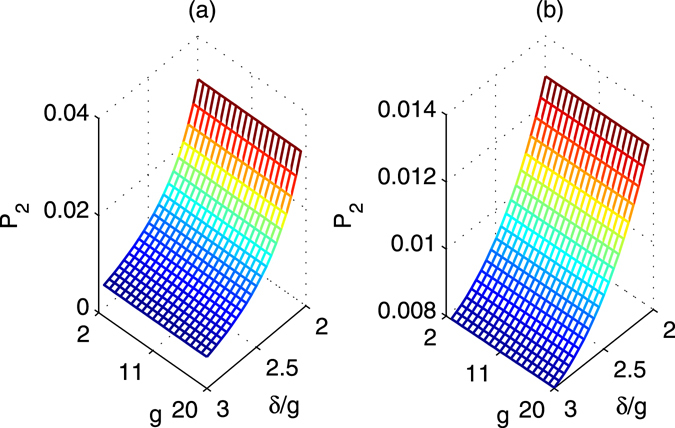
The probability *P*_2_. (**a**) The first case; (**b**) The second case.

The *P*_2_ is shown in Fig. 8 for two groups of parameters. The effective decoherence rates due to the atomic spontaneous emission and the decay of the Bosonic modes are ${\rm{\Gamma }}^{\prime} ={P}_{1}{\rm{\Gamma }} < {10}^{-4}g$ and $\kappa ^{\prime} ={P}_{2}\kappa  < 0.35\times {10}^{-3}g$, respectively.

**Figure 9:**
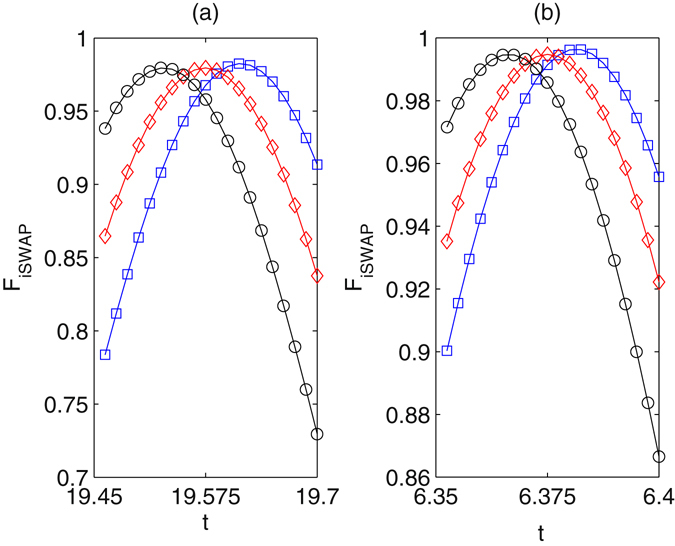
The average fidelity of the iSWAP gate vias *g* and evolution time. ${{\rm{\Delta }}}_{1}=9g$, ${{\rm{\Delta }}}_{2}=9g+\delta$, *ν* = 4*g*, ${\rm{\Omega }}=2g$. (**a**) The diamonds refer to $g\approx 5.275$, the squares and the circle refer, respectively, to a variation of −0.025 and +0.025 of *g*. (**b**) The diamonds refer to $g\approx 18.4$, the squares and the circle refer, respectively, to a variation of −0.05 and +0.05 of *g*.

**Figure 10:**
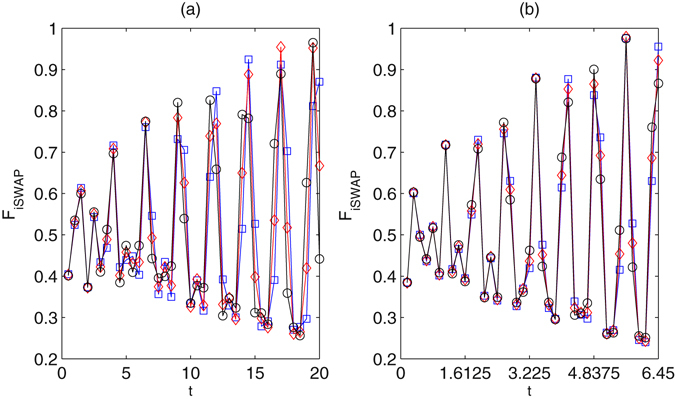
The fidelity of the iSWAP gate vias *g* and evolution time. ${{\rm{\Delta }}}_{1}=9g$, ${{\rm{\Delta }}}_{2}=9g+\delta$, *ν* = 4*g*, ${\rm{\Omega }}=2g$. (**a**) The diamonds refer to $g\approx 5.275$, the squares and the circle refer, respectively, to a variation of −0.025 and +0.025 of *g*. (**b**) The diamonds refer to $g\approx 18.4$, the squares and the circle refer, respectively, to a variation of −0.05 and +0.05 of *g*.

**Figure 11:**
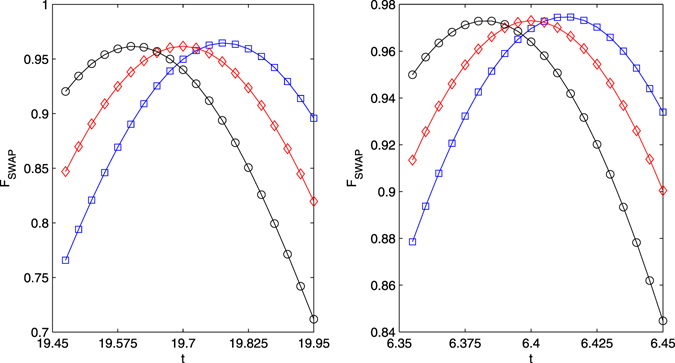
The fidelity of the iSWAP gate vias *g* and evolution time using master equation. ${{\rm{\Delta }}}_{1}=9g$, ${{\rm{\Delta }}}_{2}=9g+\delta$, *ν* = 4*g*, κ = γ = β = 0.002g. ${\rm{\Omega }}=2g$. (**a**) The diamonds refer to $g\approx 5.275$, the squares and the circle refer, respectively, to a variation of −0.025 and +0.025 of *g*. (**b**) The diamonds refer to $g\approx 18.4$, the squares and the circle refer, respectively, to a variation of −0.05 and +0.05 of *g*.

The fidelity of the iSWAP gate is defined by

29 $${F}_{iSWAP}=\int Tr[\sqrt{\sqrt{{\rho }_{o}}{\rho }_{i}\sqrt{{\rho }_{o}}}$$

over all possible states, where *ρ*_*o*_ denotes the real final density matrix while *ρ*_*i*_ denotes the ideal final density matrix. The fidelity of the iSWAP gate is shown in Fig. 9. For the small $g\approx 5.275$, the fidelity may be reached to 0.982 after the evolution time $t\approx 19.575$, see Fig. 9(a). For the large $g\approx 18.4$, the fidelity may be reached to 0.994 after the evolution time $t\approx 6.375$. The ideal iSWAP gate is achieved after eight Rabi-like oscillations, see Fig. 10. In the regime $\nu \gg {g}_{i}$ the fidelities of the gates have been consistently found to be essentially unaffected by fiber losses. In general, moreover, the direct effect of spontaneous emission proves to be more relevant than the indirect effect of cavity losses. For the iSWAP gate with $\nu \approx 4g$ and $g\approx 5.275$, the maximum fidelity drops to $F\approx 0.958$ for $\kappa =\gamma =\beta =0.002g$, see Fig. 11. If large coupling strength $g\approx 18.4$, the maximum fidelity drops to $F\approx 0.972$ for $\kappa =\gamma =\beta =0.002g$. With lower decay rates $\approx$0.0002*g* the iSWAP gate is unaffected, while it may be spoiled if high rate $\approx$0.1*g* is considered. The spontaneous emission rates should be restricted for the fabrication of high-finesse optical cavities in experiment. Hyperfine ground levels of effective high level lambda systems could be candidates for such schemes. Take ^87^Rb atoms as examples^56^. Three ground states may be defined by hyperfine atomic levels $|F=1,m=-1\rangle$, $|F=1,m=0\rangle$, $|F=1,m=1\rangle$ of 5^2^ *S*_1/2_, while excited state may be defined by the hyperfine atomic level |*F* = 1, *m* = 0〉 of 5^2^ *P*_1/2_. Each atom can be made localized at a fixed position in each cavity with high *Q* for long time^56^. Recent experiment^57^ has achieved the parameters *g*/2*π* $\approx$ 750 MHz, *κ*/2*π* $\approx$ 2.62 MHz, and *γ*/2*π* $\approx$ 3.5 MHz in an ultrahigh-*Q* toroidal microresonators with the wavelength in the region 630~850 nm is predicatively achievable with the optical fiber decay rate 0.152 MHz^58^. By setting ${{\rm{\Omega }}}_{1}={{\rm{\Omega }}}_{2}=0.35g$, Δ_1_ = 2.3*g*, Δ_2_ = 2.4*g*, and *ν* = 0.8*g*, we can obtain a iSWAP gate the fidelity about 9.21% with $\kappa \approx 0.0035g$ and $\gamma \approx 0.0046g$.

## Conclusion

In conclusion, we have investigated the implementation of high-dimensional quantum computation for atoms trapped in distant cavities coupled by an optical fiber. The chosen ground states of each atom are coupled via the cavity mode and different classical fields in the Raman process. All the atoms do not undergo the real Raman transitions due to the large detuning while the atomic system is decoupled from the cavity modes and fiber modes. In the short fiber regime, reliable elementary gates could be reasonable even if imperfections (atomic spontaneous decay and photon leakage of the cavities and fibers) are considered. Let us also mention that, in the considered system, not only entangling and swap gates, but also perfect quantum state transfer is possible. Moreover, the proposed setup would also allow for entanglement preparation schemes between distributed atoms, and could useful in one-way quantum computation. These schemes would be useful for constructing large-scale and long-distance quantum computation or quantum communication networks.

## Method

By introducing new Bosonic modes ${c}_{1}=\frac{1}{\sqrt{2}}({a}_{1}-{a}_{2})$, ${c}_{2}=\frac{1}{2}({a}_{1}+{a}_{2}+\sqrt{2}b)$ and ${c}_{3}=\frac{1}{2}({a}_{1}+{a}_{2}-\sqrt{2}b)$, the Hamiltonians *H*_0_ may be rewritten as ${H}_{0}=\frac{\nu }{\sqrt{2}}({c}_{2}^{ {\dagger } }{c}_{2}-{c}_{3}^{ {\dagger } }{c}_{3})$. Take *H*_0_ as the free Hamiltonian and perform the unitary transformation $U={e}^{i{H}_{0}t}$, it follows an efficient interaction Humiliation

30 $$\begin{array}{rcl}{H}_{{int}}^{^{\prime} } & = & \frac{{\zeta }_{{i}_{2}}}{4}\mathrm{(2}{c}_{1}^{ {\dagger } }{c}_{1}+{c}_{2}^{ {\dagger } }{c}_{2})\sum _{j=1}^{2}{|{e}_{{i}_{2}}\rangle }_{jj}\langle {e}_{{i}_{2}}|-\sum _{j=1}^{2}{\eta }_{{i}_{1}}{|{e}_{{i}_{1}}\rangle }_{jj}\langle {e}_{{i}_{1}}|\\  &  & -\,\frac{1}{4}\{2{\lambda }_{{i}_{1}{i}_{2}}[\sqrt{2}{e}^{i{\delta }_{{i}_{1}{i}_{2}}t}{c}_{1}+{e}^{i({\delta }_{{i}_{1}{i}_{2}}-\sqrt{2}\nu )t}{c}_{2}+{e}^{i({\delta }_{{i}_{1}{i}_{2}}+\sqrt{2}\nu )t}{c}_{3}]{S}_{1}^{+}\\  &  & +\,2{\lambda }_{{i}_{1}{i}_{2}}[\sqrt{2}{e}^{i{\delta }_{{i}_{1}{i}_{2}}t}{c}_{1}-{e}^{i({\delta }_{{i}_{1}{i}_{2}}-\sqrt{2}\nu )t}{c}_{2}-{e}^{i({\delta }_{{i}_{1}{i}_{2}}+\sqrt{2}\nu )t}{c}_{3}]{S}_{2}^{+}\\  &  & +\,{\zeta }_{{i}_{1}{i}_{2}}[\sqrt{2}{e}^{i\sqrt{2}\nu t}{c}_{2}^{ {\dagger } }{c}_{1}+{e}^{i\sqrt{2}\nu t}{c}_{2}^{ {\dagger } }{c}_{3}+\sqrt{2}{e}^{-i\sqrt{2}\nu t}{c}_{3}^{ {\dagger } }{c}_{1}]{|{e}_{{i}_{2}}\rangle }_{11}\langle {e}_{{i}_{2}}|\\  &  & +\,{\zeta }_{{i}_{1}{i}_{2}}[-\sqrt{2}{e}^{i\sqrt{2}\nu t}{c}_{2}^{ {\dagger } }{c}_{1}+{e}^{i\sqrt{2}\nu t}{c}_{2}^{ {\dagger } }{c}_{3}-\sqrt{2}{e}^{-i\sqrt{2}\nu t}{c}_{3}^{ {\dagger } }{c}_{1}]{|{e}_{{i}_{2}}\rangle }_{22}\langle {e}_{{i}_{2}}|\}+H{\rm{.}}c\mathrm{.}\end{array}$$

The Hamiltonian describes multiple off-resonant Raman couplings for each atom induced by the classical field and the Bosonic modes *c*_1_, *c*_2_, *c*_3_. If ${\delta }_{{i}_{1}{i}_{2}}\gg {\lambda }_{{i}_{1}{i}_{2}}$, $|{\delta }_{{i}_{1}{i}_{2}}\pm \sqrt{2}\nu |\gg {\lambda }_{{i}_{1}{i}_{2}}/2$ and $\sqrt{2}\nu \gg {\lambda }_{{i}_{1}{i}_{2}}/2$, ${\eta }_{{i}_{1}}/4$, the Bosonic modes do not exchange quantum numbers with the atomic system. The off-resonant Raman coupling leads a Stark shift between the atoms.

Thus the effective Hamiltonian is defined by

31 $$\begin{array}{rcl}{H}_{eff} & = & \frac{{\zeta }_{{i}_{2}}}{4}\mathrm{(2}{c}_{1}^{ {\dagger } }{c}_{1}+{c}_{2}^{ {\dagger } }{c}_{2})\sum _{j=1}^{2}{|{e}_{{i}_{2}}\rangle }_{jj}\langle {e}_{{i}_{2}}|-\sum _{j=1}^{2}{\eta }_{{i}_{1}}{|{e}_{{i}_{1}}\rangle }_{jj}\langle {e}_{{i}_{1}}|\\  &  & -\sum _{j=1}^{2}\frac{{\lambda }_{{i}_{1}{i}_{2}}^{2}}{4}\{[\frac{2}{{\delta }_{{i}_{1}{i}_{2}}}{c}_{1}{c}_{1}^{ {\dagger } }+\frac{1}{{\delta }_{{i}_{1}{i}_{2}}-\sqrt{2}\nu }{c}_{2}{c}_{2}^{ {\dagger } }+\frac{1}{{\delta }_{{i}_{1}{i}_{2}}+\sqrt{2}\nu }{c}_{3}{c}_{3}^{ {\dagger } }]{|{e}_{{i}_{1}}\rangle }_{jj}\langle {e}_{{i}_{1}}|\\  &  & +\,[\frac{2}{{\delta }_{{i}_{1}{i}_{2}}}{c}_{1}^{ {\dagger } }{c}_{1}-\frac{1}{{\delta }_{{i}_{1}{i}_{2}}-\sqrt{2}\nu }{c}_{2}^{ {\dagger } }{c}_{2}-\frac{1}{{\delta }_{{i}_{1}{i}_{2}}+\sqrt{2}\nu }{c}_{3}^{ {\dagger } }{c}_{3}]{|{e}_{{i}_{2}}\rangle }_{jj}\langle {e}_{{i}_{2}}|\\  &  & +\,\frac{{\zeta }_{{i}_{1}{i}_{2}}^{2}}{32\sqrt{2}\mu }({c}_{2}^{ {\dagger } }{c}_{2}-{c}_{3}^{ {\dagger } }{c}_{3})[{({|{e}_{{i}_{1}}\rangle }_{11}\langle {e}_{{i}_{1}}|+{|{e}_{{i}_{1}}\rangle }_{22}\langle {e}_{{i}_{1}}|)}^{2}\\  &  & +\,4{({|{e}_{{i}_{1}}\rangle }_{11}\langle {e}_{{i}_{1}}|-{|{e}_{{i}_{1}}\rangle }_{22}\langle {e}_{{i}_{1}}|)}^{2}]-{\chi }_{{i}_{1}{i}_{2}}({S}_{1}^{+}{S}_{2}^{-}+{S}_{1}^{-}{S}_{2}^{+})\end{array}$$

where

32 $${\chi }_{{i}_{1}{i}_{2}}=\frac{{\lambda }_{{i}_{1}{i}_{2}}^{2}}{4}[\frac{2}{{\delta }_{{i}_{1}{i}_{2}}}-\frac{2}{{\delta }_{{i}_{1}{i}_{2}}-\sqrt{2}\nu }-\frac{2}{{\delta }_{{i}_{1}{i}_{2}}+\sqrt{2}\nu }]$$

Since $2{c}_{1}^{ {\dagger } }{c}_{1}$, ${c}_{2}^{ {\dagger } }{c}_{2}$, ${c}_{3}^{ {\dagger } }{c}_{3}$ commute with the Hamiltonian *H*_*eff*_, the bosonic modes are unchanged if the vacuum states are applied.
